# Inheritance and Fitness Costs of Vip3Aa19 Resistance in *Mythimna separata*

**DOI:** 10.3390/toxins14060388

**Published:** 2022-06-02

**Authors:** Yueqin Wang, Jing Yang, Tiantao Zhang, Shuxiong Bai, Zhenying Wang, Kanglai He

**Affiliations:** 1 State Key Laboratory for Biology of Plant Diseases and Insect Pests, Institute of Plant Protection, Chinese Academy of Agricultural Sciences, Beijing 100193, China; zhtiantao@163.com (T.Z.); baishuxiong@caas.cn (S.B.); zywang@ippcaas.cn (Z.W.); hekanglai@caas.cn (K.H.); 2 Beijing Institutes of Life Sciences, Chinese Academy of Sciences, Beijing 100101, China; yangjing@biols.ac.cn

**Keywords:** *bacillus thuringiensis*, *mythimna separata*, inheritance, fitness costs, resistance management

## Abstract

The “high-dose/refuge” strategy is expected to work most effectively when resistance is inherited as a functionally recessive trait and the fitness costs associated with resistance are present. In the present study, a laboratory selected *Mythimna separata* strain that have evolved >634.5-fold resistance to Vip3Aa19 was used to determine the mode of inheritance. To determine if fitness costs were associated with the resistance, life history parameters (larva stage, pupa stage, pupal weight, adult longevity and fecundity) of resistant (RR), -susceptible (SS) and heterozygous (R_♂_S_♀_ and R_♀_S_♂_) strains on nontoxic diet were assayed. The LC_50_ values of R_♀_S_♂_ were significantly higher than that of R_♂_S_♀_ (254.58 μg/g vs. 14.75 μg/g), suggesting that maternal effects or sex linkage were present. The effective dominance *h* of F_1_ offspring decreased as concentration increased, suggesting the resistance was functionally dominant at low concentration and recessive at high concentration. The analysis of observed and expected mortality of the progeny from a backcross suggested that more than one locus is involved in conferring Vip3Aa19 resistance. The results showed that significant differences in many life history traits were observed among the four insect genotypes. In short, resistance to Vip3Aa19 in *M. separata* was inherited as maternal and multigene and the resistance in the strain was associated with significant fitness costs. The results described here provide useful information for understanding resistance evolution and for developing resistance management strategies.

## 1. Introduction

The oriental armyworm, *Mythimna separata* (Walker), (Lepidoptera: Noctuidae), is a typical long-distance migratory and polyphagous insect pest that causes considerable economic damage to numerous important agricultural crops, including maize, wheat, rice, vegetables, and which consequently poses a major threat to food security in China and other parts of Asia [1,2,3]. Due to high frequency use of chemical insecticides, the susceptibility of oriental armyworm was less sensitive to traditional chemicals, thus leading to unsatisfactory control results against oriental armyworm.

*Bacillus thuringiensis*, i.e., Bt, as well as transgenic crops engineered to express insecticidal proteins derived from Bt (Bt crops) can provide excellent control for oriental armyworm [4,5]. Since first introduced in 1996, Bt crops have been extensively adopted worldwide for the control of insect pests of maize and cotton. In 2019, global adoption of Bt crops reached over 100 million hectares [6]. However, the sustainability of transgenic technology may be compromised due to the field-evolved practical resistance by target pest species. In order to achieve the sustainable utilization of this technology, it is necessary to implement effective resistance management strategies. Currently, the high-dose/refuge approach and multi-gene strategy have been most recommended [7,8].

The concept underlying the high-dose/refuge strategy is that the rare resistant pests originating from Bt plants will mate with susceptible individuals developed from refuges of host plants that do not make Bt toxins. If resistance is inherited as a functionally recessive trait, the resulting heterozygous progeny can be killed by the high dose Bt protein expressed in Bt crops, thus reducing the frequency of resistance alleles and delaying the evolution of resistance [9,10]. In contrast, non-recessive (e.g., additive or dominant) inheritance of resistance will cause resistance to evolve more quickly [11].

Evolution of pest resistance to Bt crops could be delayed or countered by using combinations of two or more distinct toxins or other traits, such as Cry and Vip toxins, that kill the same pest [12,13]. Bt crop ‘pyramids’ are also designed to improve efficacy and broaden the spectrum of pests killed [13]. Vip3Aa, produced during vegetative stages of growth, have no structural homology and share no binding sites compared with Cry proteins, making their combination a ‘natural pyramid strategy’ [14,15,16]. In fact, Vip3Aa that produced in combination with Cry1Ab, Cry1F, or both in Bt corn and with Cry1 and Cry2A toxins in Bt cotton have already been commercialized in the United States, Brazil and Australia [12,17]. Compared with the extensive study of Cry proteins resistance during the past two decades, very few studies have been carried out so far for resistance to Vip3Aa proteins.

Theoretical mathematical models also suggests that fitness costs could play a key role in delaying resistance by selecting against resistant genotypes in the refuges where the Bt protein is not present [11,18]. Fitness costs associated with Bt resistance occur when insect genotypes conferring at least one allele of resistance have lower fitness than susceptible ones in the absence of selection pressure [11,18]. Fitness costs are detected when one or more life-history parameters such as survival, growth rate, size, or fecundity in resistant insects are impaired relative to susceptible ones or when the resistance level decreases over time in the absence of selection [19,20,21]. Therefore, accurate estimates of the fitness cost are important to understand the evolution of resistance and define resistance management measures to mitigate resistance to transgenic plants.

Our primary objective here was to evaluate the inheritance and fitness costs of *M. separata* with Vip3Aa19 resistance. Results generated from this research should provide valuable information for the sustainable use of Vip3Aa19 technology for control of *M. separata* and develop effective strategies for managing resistance.

## 2. Results

### 2.1. Inheritance of Vip3Aa19 Resistance in M. separata

After being selected for 18 generations, the LC_50_ values for SS and RR were 1.97 (1.50–2.55) μg/g and >1250 μg/g, respectively (Table 1). The difference in the LC_50_ values of the two parental strains was significant based on the non-overlapped 95% FLs, resulting in a resistance ratio of more than 634.5-fold for RR strain (Table 1). The LC_50_ values differ significantly between the F_1RS_ [254.58 (189.09–323.04) μg/g] and F_1SR_ [14.75 (9.65–20.20) μg/g] progeny of the reciprocal crosses based on the non-overlapped 95% FLs, which suggested that maternal effects and sex linkage were present in the Vip3Aa19 resistance. The LC_50_ values for the four backcross strains between F_1_ and RR ranged from 4.57 (2.94–6.36) μg/g to 27.35 (18.69–37.07) μg/g, with the resistance ratio ranged from 2.3 (1.5–3.7) to 13.9 (9.0–21.4) (Table 1).

The degrees of dominance estimated by Stone’s method were 0.68 and −0.30 for reciprocal crosses of F_1RS_ and F_1SR_, respectively. 

Based on the mortality at each Vip3Aa19 concentration in diet-incorporated bioassay, the effective dominance level, *h*, varied depended upon the F_1_ reciprocal cross and the concentration of Vip3Aa19. For F_1RS_, *h* > 0.5 at the concentrations of 5.0 μg/g to 250 μg/g, suggesting the resistance was functionally incompletely dominant. However, at the highest concentration of 625 μg/g, *h* was 0.19, indicating that the resistance was incompletely recessive. For F_1SR_, the resistance was incompletely dominant at the concentration of 5 µg/g (*h* = 0.68) and declined to incompletely recessive at 50 µg/g (*h* = 0.18). At the highest concentration of 250 μg/g and 625 μg/g, *h* was 0, indicating that resistance was completely recessive (Table 2).

The backcross of F_1RS_ with resistant parents had higher actual mortality than expected values resulting from all of the four Vip3Aa19 toxin doses in a series. Moreover, when ∑χ^2^ was used, the monogenic hypothesis was rejected (∑χ^2^ > ∑χ^2^ _0.05_ = 3.84, df = 1), indicating that more than one locus is involved in conferring resistance (Table 3).

### 2.2. Fitness Costs of Vip3Aa19 Resistance in M. separata

On non-Bt diet, the effect of insect colony on larva stage and pupation rate were significant, while it was not significant for all other life history parameters such as pupa stage, pupal weight and male adult longevity. The average larval developmental time was significantly longer for RR and F_1RS_ compared to SS and F_1SR_, which were 29.2 d and 28.5 d, respectively. On average, the susceptible and F_1SR_ individual both needed 27.7 d to develop to the pupal stage. The pupation rate was significantly lower for Vip3Aa19 resistant strains relative to the susceptible and two reciprocal crosses. However, progeny from reciprocal crosses were not significantly different from the susceptible strain (Table 4 and Table 5).

The number of eggs per female of the two F_1_ hybrid colonies strains were 1054.2 and 983.9, respectively. The number of eggs per female of SS (1193.8) was not significantly different from F_1_ hybrid colonies, while it was significantly greater than the number of RR (847.9). SS and F_1_ hybrid colonies strains had a similar emergence rate, ranged from 70.9% to 80.3%, but the rate of larvae developed to adult of RR strain was significantly declined to 63.7%. The preoviposition stage and female adult longevity were significantly reduced in F_1RS_ strain compared with the RR and F_1SR_ strains, but no significant differences were detected between F_1RS_ and SS. Among the types tested, no significant differences were found in the male adult longevity (Table 5).

Fertility life table parameters including the mean generation time (*T*), the net reproductive rate (*R*_0_) and the intrinsic rate of increase (*r_m_*) presented different values between the homozygous parent strains and reciprocal crosses, except for the finite rate of increase (*λ*). *T* of the RR strain were significantly longer than this of SS and heterozygotes derived from the RR_♀_ × SS_♂_cross. However, *T* was numerically longer for RR than for F_1SR_ but the difference was not statistically significant (Table 6).

## 3. Discussion

Knowledge of the genetic basis of resistance to Bt toxins or Bt crops is important for evaluating the risk of resistance and designing effective resistance management strategies. In this study, we estimate the pattern of inheritance of resistance to Vip3Aa19 in a lab-selected *M. separata* strain, which was originated from the SS population. These significant lower LC_50_ value of the F_1_ progeny from SS female × RR male cross compared with the RR female × SS male cross indicated that resistance to Vip3Aa19 was inherited with maternal effects rather than autosomal. The degree of dominance of F_1_ reciprocal cross at LC_50_ level (−0.30 vs. 0.68) gives further evidence of a possible maternal influence on Vip3Aa19 resistance. Maternal influences have been found in populations of *Plutella xylostella* (Lepidoptera: Plutellidae) to Cry1Ac and Cry1Ab [22,23,24]. However, there is a report where the gender of the resistant parents had a strong paternal influence on Vip3Aa resistance in *Heliothis virescens* (Lepidoptera: Noctuidae) [25]. With the possible parental influence on resistance, reduced mating success may help to limit an increase in the frequency of the resistant allele, thus contribute to delays the evolution of resistance. Many studies suggested that autosomal inheritance of resistance is common in numerous lepidopterous insects, including *P. xylostella* to Cry2Ad [26], *Ostrinia furnacalis* (Lepidoptera: Crambidae) to Cry1F and Cry1Ah [27,28], *M. unipuncta* (Lepidoptera: Noctuidae) to Cry1Ab [29], *Helicoverpa zea* (Lepidoptera: Noctuidae) to Cry2Ab2 [30], *Spodoptera frugiperda* (Lepidoptera: Noctuidae) to Cry2Ab2 and Vip3Aa20 [21,31]. These significant differences demonstrate that it is vital demand for understanding species-specific genetic model of resistance to program case specific resistance management strategy.

The degree of dominance of Vip3Aa19 resistance in *M. separata* depended on the tested concentration and the F_1_ reciprocal cross. The dominance was incompletely dominant in resistant females crossed with susceptible males at 50 μg/g and 250 μg/g, whereas it was incompletely recessive or complete recessive in susceptible females crossed with resistant males at two toxin concentrations. In general, the degree of dominance decreased with increasing toxin concentration, with resistance being nearly recessive at a high concentration [26,30]. Greenhouse tests indicated that dominance may vary depending on different levels of Bt toxin expression in tissues during different plant stages (e.g., vegetative-stage and reproductive-stage). For *O. furnacalis*, the resistance to *cry1Ie*-maize is nearly completely recessive on Cry1Ie maize leaf, but nearly completely dominant on Cry1Ie maize silk [32]. This suggests that a high dose in transgenic maize is necessary for a refuge strategy to be successful in delaying resistance [30]. If resistance is recessive, the offspring from mating between resistant and susceptible insects will die on high-dose Bt crops, substantially delaying the evolution of resistance [10]. When recessive resistance appears not to be achieved, increasing refuge abundance can still substantially delay resistance [10]. For example, modeling results suggest that resistance can be delayed for >20 years with ≥5% refuges if resistance is completely recessive (*h* = 0) and with >50% refuges if resistance is partially dominant (*h* ≥ 0.4) [33].

In the present study, the method used to determine the number of loci involved in resistance is based on the expected mortality data from the backcross at each toxin dose. The test of monogenic mode did not fit the data, revealing that more than one locus (polygenic) was involved in resistance. The resistance mechanisms of *M. separata* to Vip3Aa may be account for this result. Quan et al. [34] reported that the resistance to Vip3Aa is not related to altered binding to midgut receptors, while altered binding to membrane receptors was the main mechanism of resistance to Cry proteins. Polygenic resistance against Cry1, Cry2 and Vip3Aa protein has also been reported in a number of insect species [25,35,36]. For example, resistance to Cry1Ab is characterized as polygenic in Cry1Ab selected strains of *O. furnacalis* [37]. More than one locus was responsible for the resistance to Cry2Ab2 in *H. zea* [30]. Pickett et al. [25] reported that resistance to Vip3Aa in lab-selected populations of *H. virescens* was due to more than one locus. Nevertheless, some populations resistant to Cry toxins have been shown to exhibit monogenic resistance, including *O. nubilalis* to Cry1Ab [38], *H. armigera* to Cry2Ab [39], *P. xylostella* to Cry1Ac [40]. The number of genes contributing to resistance appears to be different in different insect species and may even differ for individual toxins and under different selection regimes [41,42]. Besides, the number of loci engaged in resistance may vary for the resistance level and genetic background [30].

Another additional factor predicted to delay resistance is fitness costs. Fitness costs occur when fitness on non-Bt host plants is lower for resistant insects than susceptible insects, so that refuges select against resistance [11,18]. At the individual level, RR strain had lower reproductive rate on non-Bt diet but had longer larval time and preoviposition time of female compared to SS strain. At the population level in terms of survival (pupation and emergence rates) rate, the net reproductive rate (*R*_0_) (F = 8.3; df = 3, 36; *p* < 0.01) and the intrinsic rate of increase (*r_m_*) (F = 15.9; df = 3, 36; *p* < 0.01), RR strain was low compared to SS strain. These results suggest that the fitness costs of resistance to Vip3Aa19 in *M. separata* are recessive traits. However, reciprocal F_1_ progenies had those biological parameters close to SS train, which indicated an absence of relevant fitness costs in heterozygous individuals, most likely due to heterosis effect. Other than *M. separata*, fitness costs of Vip3A resistance have only been reported in *S. frugiperda* and *H. virescens* [31,43]. Bernardi et al. [31] observed the presence of relevant fitness costs of Vip3A resistance in *S. frugiperda*, which had a lower survival and reproductive rate on non-Bt corn leaf compared with susceptible and heterozygous strains. However, Chen et al. [44] observed no fitness costs of Vip3A resistance in *S. frugiperda* on either the meridic diet or non-Bt corn leaves. The differences in the results may be due to the diverse genetic variations in the resistant populations or the different assay methods and test substances [18,45]. A substantial fitness costs were present in a Vip3A-resistant colony of *H. virescens*, which showed reduced survival to adult eclosion, lower egg viability and mating success [43].

Fitness costs can delay resistance evolution by selecting against Bt-resistant genotypes or in refuges where insects are not exposed to Bt toxins. A lack of fitness costs can favor the persistence of alleles that are responsible for resistance when Bt toxin is absent, potentially maintaining the frequency of resistant individuals in the field and contributing to the rapid development of resistance to Bt crops. Previous publications have shown that field-evolved resistance to Bt crops is associated with the lack of fitness costs in several cases, including *Busseola fusca* (Lepidoptera: Noctuidae) to Cry1Ab corn in South Africa [46], *S. frugiperda* to Cry1F corn in Puerto Rico [47], *Diabrotica virgifera virgifera* (Coleoptera: Chrysomelidae) to Cry3Bb1 corn in the United States [48]. The magnitude and dominance of fitness costs can be affected by ecological factors such as host-plant cultivar or species, entomopathogen, insect pathogens, and fitness costs seem to be greater under stressful conditions [49,50]. For example, Raymond et al. [51] reported that fitness costs were higher on the low-quality host, with the magnitude of fitness costs increasing with reduced suitability of host. However, laboratory bioassays data generated by Chen et al. [44] showed that lower suitability and plant phytochemicals do not increase the magnitude or dominance of fitness costs associated with Bt resistance. Therefore, the evaluation of the interactions between non-Bt hosts and fitness costs should be case-by-case.

## 4. Conclusions

In summary, the lab-selected *M. separata* have developed a high level of resistance to Vip3Aa19 and offered a valuable opportunity to characterize the genetic basis and fitness costs of resistance. The maternal and multigene trait of inheritance and the presence of fitness costs indicate favorable conditions for resistance management. In the future, laboratory bioassays evaluating the interactions between non-Bt refuge host plants and fitness costs of Vip3Aa resistance could be carried out. 

## 5. Materials and Methods

### 5.1. Insect Strains

A field population of *M. separata* was collected from farmers’ fields in Gongzhuling, Jilin Province, where Bt corn is not grown. The field population was divided into two subpopulations at the egg stage of the third generation. One was left unselected and was reared on artificial diet based on powdered corn leaf and wheat germ in the laboratory for 20 generations without exposure to Bt toxins [52]. This strain was designed as SS. The other one was selected with Vip3Aa19 at the 1st instar larval stage from the 3rd generation (RR strain). The selection was at a concentration that would generate 95–99% mortality based on the results of previous bioassays [52]. After 7 days of exposure, survivors were transferred to a container containing Bt-free artificial diet for the next generation. Resulting pupae were transferred to mating cages and a cotton ball soaked with 10% honey (*v*/*v*) water solution was provided. Waxed paper as an egg depositing substrate was placed in the cage and collected daily. All egg masses were stored at 4 °C until used. All stains were incubated at 24 ± 1 °C with a 16L: 8D photoperiod and 60% relative humidity. 

The resistance characteristics were tested at the 18th generation.

### 5.2. Bt Toxins

Vip3Aa19 was obtained by IPTG (Isopropyl-beta-D-thiogalactopyranoside) induction and affinity column chromatography (GE Healthcare) and stored at −80 °C.

### 5.3. Susceptibility Bioassays

The susceptibility of neonates to Bt toxins was determined in survival bioassays by exposing neonate larvae (<12 h after hatching) to a series of concentrations of purified toxin and a negative control (only water applied to the diet) incorporated into the agar-free semi-artificial diet [53]. Neonate larvae were transferred to individual wells in 24-well plastic trays. Bioassays were repeated two times to give 48 neonates per test concentration and included 9–13 concentrations. The trays were covered with membrane (Cat# 3M-9733, Minnesota Mining and Manufacturing Company, Saint Paul, Minnesota (MN), USA) and maintained at 24 ± 1 °C with a photoperiod of 16: 8 h (L: D) and 60% RH. The number and weight of survivors per treatment were recorded 7 days following infestation. If a larva had not developed beyond the first instar and weighed ≤0.2 mg, it would be counted as dead [53]. The toxicity of Vip3Aa19 to different insect strains or genetic crosses was analyzed by probit regression using PoloPlus (LeOra Software). 

### 5.4. Genetic Crosses

To determine the inheritance of Vip3Aa19 resistance in *M. separata*, two types of crosses were performed to generate six additional insect colonies. Reciprocal crosses were performed between the parental resistant strain (RR) and susceptible strain (SS) to establish two lines: F_1RS_ (RR_♀_ × SS_♂_) and F_1SR_ (SS_♀_ × RR_♂_) [54] (Liu and Tabashnik, 1997). The backcross lines (BC) were obtained from the reciprocal progenies of the F1 adults cross with adults from the parental strain (RR): BC_1_ (F_1RS__♀_ × RR_♂_), BC_2_ (RR_♀_ × F_1RS__♂_), BC_3_ (F_1SR__♀_ × RR_♂_), BC_4_ (RR_♀_ × F_1SR__♂_). Twenty unmated males and females were used for each cross and backcross. Those mass crosses provided enough offspring for following multiple concentration testing and calculation of LC_50_ values.

### 5.5. Maternal Effects or Sex-Linkage

Maternal effects or sex-linkage of resistance was evaluated by comparing the LC_50_ values of F_1_ hybrid progenies derived from the reciprocal crosses between parental strains. If LC_50_ values of the two F_1_ reciprocal crosses are significantly different, resistance is regarded as sex-linked, otherwise the resistance is regarded as autosomal [54].

### 5.6. Estimation of Degree of Dominance

The degree of dominance (*D*) was estimated on the basis of LC_50_ values of F_1_ progeny from reciprocal crosses according to Stone’s method [55]:

*D* = (2X_2_ − X_1_ − X_3_)/(X_1_ − X_3_) (1)

where X_1_, X_2_ and X_3_ are the logLC_50_ values of RR, F_1_ and SS strains, respectively. *D* varies from −1 (completely recessive) to 1 (completely dominant), with 0 indicating either incomplete dominance or additivity. 

The effective dominance (*h*) was calculated based on the survival at any single concentration of the RR, SS and their F_1_ crosses according to equation described in Bourguet et al [56].

*h* = (W_RS_ − W_SS_)/(W_RR_ − W_SS_) (2)

where W_RS_, W_SS_ and W_RR_ are the fitness values at a given concentration for heterozygotes, susceptible and resistant homozygotes, respectively. W_RR_ was assumed to be 1 at any treatment concentration. The fitness of the susceptible parent and the heterozygous F_1_ was estimated from the survival rate of the larvae at a specific treatment concentration divided by the survival rate of the resistant parent at the same concentration. The resulting *h* varies from 0 (completely recessive resistance) to 1 (completely dominant resistance), with 0.5 indicating semi-dominant or codominant.

### 5.7. Number of Loci Influencing the Inheritance

Monogenic resistance or multiple-gene resistance was estimated by the chi-square (χ^2^) test and concentration-response curve goodness-of-fit between observed and expected mortalities of the backcross population at different concentrations [57,58]. The null hypothesis is that resistance is controlled by one locus with two alleles, S (susceptible) and R (resistant). If so, the backcross between RS and RR will produce progeny that are 50% RR and 50% RS. *p_i_*, the expected mortality in the backcross progeny at concentration *i*, is calculated as

*p_i_* = 0.5 (*M*_RS_ + *M*_RR_) (3)

where *M*_RS_ and *M*_RR_ are the mortalities of the presumed RS and RR genotypes at concentration *i*, respectively. 

The χ^2^ values were then calculated for as follows:

χ^2^ = (*F_i_* − *p_i_n*)^2^/*p_i_qn* (4)

Observed mortality *O_BC_* in the backcross progeny at concentration *i*, is calculated as

*O_BC_* = *F_i_*/*n* (5)

where *F_i_* is the observed number of dead larvae in backcross survival bioassays at dose *i*, *n* is the number of backcross progeny exposed at dose *i*, *p_i_* is the expected mortality, and *q* = *1* − *p_i_*_._ Then the sum of χ^2^ (∑χ^2^) at each concentration was compared with a χ^2^ distribution with one degree of freedom. The null hypothesis of monogenic resistance could be accepted if ∑χ^2^ < χ^2^ _0.05_ (df = 1), otherwise the resistance is controlled by multiple genes [57,58].

### 5.8. Assessing Fitness Costs of Vip3Aa19 Resistance

Fitness costs associated with the Vip3Aa19 resistance in *M. separata* were evaluated on non-Bt meridic diet. Neonates (<12 h after hatching) from RR, SS, R_♂_S_♀_, and R_♀_S_♂_ were placed individually on non-Bt diet. Bioassays were conducted in 24-well bioassay trays. For each strain, 72 larvae in total were tested. The trays were held in an environmental at 24 ± 1 °C, 60% RH under a 16: 8 h light/dark photoperiod. Fresh artificial diet was replaced when necessary until insect pupation. The following biological parameters were recorded: duration of egg, larval and pupal periods, pupal weight, sex ratio, preoviposition stage, adult longevity, fecundity, the total cycle duration. Pupal weight and sex were assessed within 24 h of pupation.

To assess fecundity, ten pairs of newly emerged (<24 h old) virgin female and male adults from the same genotype were placed into the mating container (12 cm height × 5 cm diameter) and maintained as described above. A plug of cotton wool saturated with 10% honey solution was placed on each container to feed adults. The number of eggs produced per female was estimated daily until the end of the oviposition period. Biological parameters and life table parameters, including the mean generation time (*T*), the net reproductive rate (number of females produced per parental female, *R_o_*), intrinsic rate of population increase (*r_m_*) and finite rate of increase (*λ*), were analyzed using TWOSEX-MSChart program. Fitness costs are present when one or more fitness components are significantly lower in the resistant strain than in the susceptible strain.

## Figures and Tables

**Table 1:** Toxicity of Vip3Aa19 to resistant strain, susceptible strain, their F_1_ progenies and back-cross progenies of *M. separata*.

Colony	n	LC_50_ (95% FL) μg/g	RR (95% FL)	Slope ± SE	χ^2^	df (χ^2^)	*D*	*h*
SS	384	1.97 (1.50–2.55)	1.0 (0.7–1.5)	1.33 ± 0.12	9.1	14		
RR	624	>1250	>634.5					
Reciprocal crosses								
F_1RS_	576	254.58 (189.09–323.04)	129.4 (89.1–187.9)	2.31 ± 0.34	7.9	22	0.68	0.84
F_1SR_	480	14.75 (9.65–20.20)	7.5 (4.8–11.7)	1.71 ± 0.24	7.4	18	−0.30	0.35
Backcrosses								
BC_1_	432	7.61 (5.75–10.01)	3.9 (2.6–5.7)	1.23 ± 0.10	10.6	16		
BC_2_	528	27.35 (18.69–37.07)	13.9 (9.0–21.4)	1.58 ± 0.19	7.7	20		
BC_3_	384	4.57 (2.94–6.36)	2.3 (1.5–3.7)	1.57 ± 0.20	9.3	14		
BC_4_	480	20.18 (14.98–25.87)	10.3 (7.0–15.0)	2.06 ± 0.25	4.3	18		

n, number of tested larvae. FL, fiducial limits. BC_1_ (F_1RS__♀_ × RR_♂_). BC_2_ (RR_♀_ × F_1RS__♂_). BC_3_ (F_1SR__♀_ × RR_♂_). BC_4_ (RR_♀_ × F_1SR__♂_).

**Table 2:** Effective of dominance (*h*) of resistance to Vip3Aa19 in *M. separata*.

Concentration (μg/g)	Colony	Survival Rate (%) ^a^	Fitness	*h*
5.0	SS	31.25	0.35	
	RR	89.58	1	
	F_1RS_	85.42	0.95	0.93
	F_1SR_	70.83	0.79	0.68
50.0	SS	2.08	0.02	
	RR	93.75	1	
	F_1RS_	81.25	0.87	0.86
	F_1SR_	18.75	0.20	0.18
250.0	SS	0	0	
	RR	87.50	1	
	F_1RS_	45.83	0.52	0.52
	F_1SR_	0	0	0
625.0	SS	0	0	
	RR	89.58	1	
	F_1RS_	16.67	0.19	0.19
	F_1SR_	0	0	0

^a^: calculated based on the survival number after being exposed to Bt toxins for 7 days.

**Table 3:** Test of monogenic mode of inheritance of resistance to Vip3Aa19 in *M. separata*.

Colony	Concentration (μg/g)	Observed Mortality (%)	Expected Mortality (%)	χ^2^
BC_1_	5.0	37.50	23.96	4.83
	25.0	70.83	38.54	21.13
	50.0	85.42	45.83	30.29
	250.0	100	56.25	37.33
∑χ^2^				93.59
BC_2_	5.0	20.83	15.63	0.99
	25.0	45.83	26.04	9.76
	50.0	68.75	37.50	20.00
	250.0	95.83	54.17	33.57
∑χ^2^				64.32

**Table 4:** Larva stage, pupa stage, pupation rate, pupal weight of susceptible strain, resistant strain, and two reciprocal F_1_ colonies of *M. separata* on non-Bt diet.

Colony	Larva Stage (d)	Pupa Stage (d)	Pupation Rate (%)	Female Pupal Weight (mg)	Male Pupal Weight (mg)
SS	27.7 ± 0.3 b	10.9 ± 0.2	84.7 ± 1.4 a	386.6 ± 8.9	376.4 ± 8.4
RR	29.2 ± 0.8 a	11.5 ± 0.4	61.2 ± 3.6 b	367.9 ± 9.3	374.9 ± 8.5
F_1RS_	28.5 ± 0.2 a	11.4 ± 0.3	75.0 ± 2.4 a	396.4 ± 7.2	384.2 ± 7.3
F_1SR_	27.7 ± 0.3 b	11.6 ± 0.3	77.8 ± 3.7 a	391.1 ± 5.8	381.9 ± 8.2
F	16.2	3.1	33.9	1.2	0.23
df	3, 211	3, 157	3, 8	3, 107	3, 100
*p*	0.000	0.027	0.000	0.313	0.837

Data are mean ± SE. Different lowercase letters in the same column indicate significant difference at *p* < 0.05 level by Tukey’s HSD test. The same below.

**Table 5:** Emergence rate, preoviposition stage, adult longevity, fecundity, one generation time of susceptible strain, resistant strain, and two reciprocal F_1_ colonies of *M. separata* on non-Bt diet.

Colony	Emergence Rate (%)	Preoviposition Stage (d)	Female Adult Longevity (d)	Male Adult Longevity (d)	Egg Laid per Female	One Generation Time (d)
SS	80.3 ± 0.3 a	5.6 ± 0.3 ab	12.3 ± 0.4	12.4 ± 0.4	1193.8 ± 149.9 a	56.0 ± 0.5 c
RR	63.7 ± 5.1 b	6.2 ± 0.3 a	12.6 ± 0.2	13.3 ± 0.4	847.9 ± 88.4 b	59.5 ± 0.6 a
F_1RS_	70.9 ± 5.0 a	5.1 ± 0.2 b	11.5 ± 0.3	13.3 ± 0.4	1054.2 ± 129.7 ab	57.6 ± 0.5 b
F_1SR_	77.1 ± 3.7 a	6.1 ± 0.2 a	12.5 ± 0.3	13.4 ± 0.4	983.9 ± 132.7ab	57.6 ± 0.6 b
F	25.8	3.1	1.2	1.4	1.1	4.7
df	3, 8	3, 100	3, 78	3, 75	3, 36	3, 36
*p*	0.000	0.038	0.327	0.245	0.037	0.007

**Table 6:** Population growth rates of *M. separata* strains susceptible or resistant to Vip3Aa19 and their F_1_ progenies.

Strain	Mean Generation Time *T* (d)	Net Reproductive Rate *R*_0_	Intrinsic Rate of Population Increase *r_m_*	Finite Rate of Pop Increase *λ*
SS	51.39 ± 0.46 b	221.07 ± 68.44 a	0.105 ± 0.006 a	1.11 ± 0.007
RR	54.32 ± 0.64 a	113.89 ± 37.05 b	0.087 ± 0.006 c	1.09 ± 0.007
F_1RS_	51.76 ± 0.84 b	178.68 ± 55.53 b	0.100 ± 0.007 b	1.10 ± 0.007
F_1SR_	52.71 ± 0.85 ab	161.29 ± 51.01 b	0.096 ± 0.007 bc	1.10 ± 0.008
F	20.4	8.3	15.9	15.8
df	3, 36	3, 36	3, 36	3, 36
*p*	<0.01	<0.01	<0.01	0.218

## Data Availability

Not applicable.
